# Goal setting in later life: an international comparison of older adults’ defined goals

**DOI:** 10.1186/s12877-024-05017-x

**Published:** 2024-05-21

**Authors:** Elissa Burton, Jill Chonody, Barbra Teater, Sabretta Alford

**Affiliations:** 1 enAble Institute, Faculty of Health Sciences, Curtin University, GPO Box U1987, Perth, WA Australia; 2 Curtin School of Allied Health, Faculty of Health Sciences, Curtin University, Perth, WA Australia; 3 College of Health Sciences, School of Social Work, Boise State University, Boise, ID USA; 4 College of Staten Island, Department of Social Work, City University of New York, Staten Island, NY USA; 5 The Graduate Center, PhD in Social Welfare, City University of New York, New York, NY USA

**Keywords:** Goals, Aging, Comparative research, Multi-country

## Abstract

**Background:**

Studies of goal setting in later life tend to focus on health-related goal setting, are pre-determined by the researcher (i.e., tick box), and/or are focused on a specific geographical area (i.e., one country). This study sought to understand broader, long-term goals from the perspective of older adults (65 + years) from Australia, New Zealand (NZ), United Kingdom (UK), Ireland, Canada, and the United States of America (USA).

**Methods:**

Through a cross-sectional, online survey (*N* = 1,551), this exploratory study examined the qualitative goal content of older adults. Thematic analysis was used to analyze the qualitative data, and bivariate analyses were used to compare thematic differences between regions and by participants’ sex.

**Results:**

Over 60% of the participants reported setting goals, and participants from the Australia-NZ and Canada-USA regions were more likely to set goals than the UK-Ireland region. The following six overarching themes were identified from the 946 goals reported: health and well-being; social connections and engagement; activities and experiences; finance and employment; home and lifestyle; and attitude to life.

**Conclusions:**

This study supports previous research that demonstrates that older adults can and do set personal goals that are wide ranging. These findings support the need for health professionals to consider different methods for elucidating this important information from older adults that builds rapport and focuses on aspects viewed as more important by the older adult and therefore potentially produces improved health outcomes.

## Background

Ageist assumptions likely influence gerontological health care workers’ perceptions of personal goal setting in older people in that they may falsely believe that they are not intent on pursuing goals beyond those related to functional abilities (e.g., improved mobility) [1]. However, “new paradigms are needed to provide guidance and support as adults move into the later decades of life, strive to maintain their independence, intent upon aging in place” [2, p.205]. This change in perspective is consistent with the World Health Organization’s (WHO) Active Aging Policy, which was developed to address the global growth in older adults and to facilitate and grow social and community connection [3]. “Active,” from the WHO’s position, is related to ongoing participation and was not meant to singularly describe health maintenance [4]. The aging process is influenced by six determinants—social, biological, behavioral, personal, health and social services, and economic—that act in concert to create individual experiences of growing older. Gender and culture are “cross-cutting determinants” given that each contributes to greater barriers and privileges. Moreover, aging is reframed as a life-long process, not something that happens at a predetermined age [4]. This framework directly informs a new paradigm of personal goal setting with its emphasis on self-determination that empowers individual decision making and inclusivity across the active aging spectrum.

Self-Determination Theory is “an empirically derived theory of human motivation and personality in social contexts that differentiates motivation in terms of being autonomous and controlled” [5, p.416]. This theory emphasizes not just the need for goals, but also the content of one’s goals [6, 7]. That is, goals may have an intrinsic motivation, such as personal growth, or health. On the other hand, goals may represent extrinsic content, such as social recognition, or financial success, which focuses on the positive evaluation of others [7, 8]. Goal content is important given its association with subjective well-being. Those who are intrinsically motivated have greater well-being than those that are extrinsically motivated, which is associated with ill-being in at least some studies (e.g [6, 7]).

Many contemporary studies address goal setting in later adult life stages; however, these studies tend to focus on health-related goal setting, such as care goals for serious illness (e.g., Ouchi, George [9]), health care preferences (e.g., Tinetti, Costello [10]) or diabetes (e.g., Kalyani, Golden [11]). Studies on personal goal setting are often dated (10 + years), and as the Baby Boomers enter older adulthood, shifts in personal goal setting is likely. The “me generation” may expect that choices are always readily available [12] and set their goals accordingly.

Personal goals have an important influence on life satisfaction and are often studied within this context. In a Canadian study of older adults, participants who had many future goals had less regret, which in turn supported a better quality of life [11]. Future goals also predicted greater life satisfaction for older people [13] as well as health and quality of life for frail older adults [14]. When examining goal content, older adults placed greater importance on intrinsic goals which were positively associated with well-being, whereas extrinsic goals were not [15].

Furthermore, studies exploring goal setting by older adults tend to be researcher driven in that pre-determined goals are provided and then rated for degree of importance, which creates a lacuna of research from the perspective of older people. For example, A study of older people in Hong Kong sought to understand differences for the young-old (i.e., 54–73 years old) and old-old (i.e., 74 years and up; Au, Ng [16]) with the six-domains of the “Goals Questionnaire Success Subscale” (as cited in Au et al. [16]). Thus, limiting respondents’ goals to these areas.

In addition, personal goal setting studies are typically conducted in a single country [13–18]. However, multi-country studies shed light on how culture may play a role and thus inform intervention development. Thus, there is much still to learn about personal goal setting by older adults. This study sought to address these gaps by elevating the voices of older people residing in Australia, New Zealand (NZ), the United Kingdom (UK), Ireland, Canada and the United States of America (USA) by initially identifying if they set goals, and for those that did, qualitatively examining their personal goals.

## Methods

### Design

This was a cross-sectional, exploratory study, distributed by online survey (using Qualtrics). This study on older adults’ goals was part of a larger survey that included questions about successful aging, living a long life, where older adults would like to live as they age, and what they need to do to stay living in their home for the rest of their life. The current analyses focuses on older adults’ goals. This study received ethical approval from [Curtin] University (HRE-2021-0587). The first question of the survey required participants to provide consent to being involved. Those who said no to giving consent were not permitted to continue completing the survey.

### Setting and sample

Prior to the distribution of the survey, a pilot test was conducted with four older adults. Two were Australians living in a regional area, one was Australian living in the suburbs surrounding a capital city and one was Scottish. Changes suggested by the pilot participants were made, such as asking age rather than birth year. At the conclusion of the pilot study, the survey was sent out to older adults through multiple channels including: (1) to older adults who previously agreed to be included in a research participant database; (2) a link in the Council of the Ageing Western Australia newsletter and the Strength for Life newsletter; (3) an advert on the Injury Matters website; (4) multiple Facebook adverts that included Australia, NZ, the UK, Ireland, Canada and the USA; and (5) multiple X (formerly twitter) posts, posted from Australia and the USA accounts. These countries were included because they were all English speaking, included at least two countries within a region of the world, and had large Facebook memberships, which allowed a large sample of people to be surveyed. The survey was distributed, and data were collected between September 2021 until April 2022.

Participants were eligible to participate in the study if they were 65 years or older, able to understand and communicate in English, and had access to the internet and/or social media. If a person did not meet these criteria, they were unable to complete the survey. Sample size calculations were based on older populations (i.e., 65 years and over) of the three regions participating in the study: (1) Australia and NZ, (2) the UK (i.e., England, Northern Ireland, Scotland, Wales) and Ireland, and (3) Canada and the USA. Considering a confidence interval of 95% and a 5% margin of error, a sample size of at least 385 survey completions was required for each region.

### Instrumentation

Participants were asked to complete demographic questions that included age, sex, where they lived (i.e. capital city and surrounding suburbs; regional city; regional area (i.e. small town, farming, etc.) or remote), who they lived with, education level, employment status, number of children, number of prescribed medications and ability to complete activities of daily living such as going to the toilet, completing own shower or bath and dressing (i.e., no difficulty, some difficulty, moderate difficulty, need help). They were also asked to complete the following questions: “Have you set goals for yourself that you would like to achieve?” (yes/no). Those who answered yes, were then asked, “If you are happy to share, can you please describe what goals you are trying to achieve currently?” (open-ended). This reduced the opportunity for the researchers to influence their answers.

Additionally, a back button was also not included on the survey. As the pilot study participants moved through the survey and were asked different questions, they explained how they thought of additional answers to previous questions due to these new questions. The researchers did not want the participants’ previous answers to be led by these new questions and therefore did not allow the back button to be included in this final, distributed survey. No incentive was offered for completing the survey.

### Data analysis

Participant demographic data were checked for normality of distribution and summarized using means and standard deviations for continuous data and frequency distribution for categorical data or non-parametric tests where required. *T*-tests, ANOVAs and chi-square tests were used for continuous and categorical group comparisons, respectively. SPSS v27 was used for data analysis, and significance levels were set at alpha = 0.05.

Qualitative data (i.e., open ended questions) were initially analyzed using thematic analysis [19, 20]. Familiarization of the data was essential, and the data set was read multiple times prior to commencing analysis. Codes were then generated initially by one researcher (EB) across the entire data set, with many codes interconnected between each other. Codes were then developed into potential sub-themes and were reviewed and discussed amongst the four authors (EB, BT, JC, SA), these sub-themes were then reviewed multiple times, and themes were generated that encapsulated a number of sub-themes within each. These themes were then reviewed and discussed by the research team (EB, BT, JC, SA) and final names of themes were agreed. A thematic map was then developed illustrating how the sub-themes were placed within themes. Given the large number of survey participants involved, after completing the thematic analysis, (multiple) themes and sub-themes were then aligned to each participant in SPSS based on their answers, to allow for further analysis between sexes and regions. For example, ID1471 reported their goals were “*Stay active Travel Be open minded Listen to grandchildren Express gratitude.”* In SPSS, ID1471’s answers were added to over-arching theme columns: Health and wellbeing; Social connection and engagement; Activities and experiences; and Attitude to life. These answers were then added to the sub-themes columns in SPSS, and for this participant included Exercise/physical activity/get fitter; Travel; Being positive and open; Spending time with family (or help them); Be kind-happy-respectful. This process was completed for each participant by the lead researcher and then checked by two authors (BT, JC).

## Results

### Study participants

Fifteen hundred and fifty-one participants completed the online survey. The mean age was 72.6 (± 5.7) years, and three-quarters were female. A third (32.5%, *n* = 530) of participants were born in the UK, 20.1% (*n* = 327) in Australia, 17.1% (*n* = 279) in Canada, 8.9% (*n* = 145) in the USA, 6.6% (*n* = 108) in NZ, and 6.7% (*n* = 110) in Ireland. Country they were living in when they completed the survey included Australia, 29.2% (*n* = 479), UK, 21.3% (*n* = 350), Canada, 20.9% (*n* = 342), USA, 7.6% (*n* = 124), Ireland, 7.9% (*n* = 129), New Zealand, 7.4% (*n* = 122). The full demographics data for the participants including a breakdown for each of the three regions: Australia/NZ; UK, Ireland and Europe; and Canada and the USA are in Table 1. The number of participants from Europe was very small (*n* = 5, Italy = 4, Portugal = 1) and were therefore combined into the UK and Ireland region.

**Table 1:** Participant demographics across the regions

Variables	Australia & NZ	UK & Ireland	Canada & USA	Total
Number participants	601	484	466	1,551
Number of goals Goals mean per person (SD)	683* 1.66 (1.78)	552 1.12 (1.50)	538* 1.42 (1.69)	1,773 1.42 (1.68)
Age M(SD) Range	72.9 (5.7) 65–99	71.5 (5.0)* 65–89	73.4 (6.2)* 65–95	72.6 (5.7) 65–99
#Sex % (n)				
- Female - Male - Non-binary - Transgender - Intersex - I prefer not to say	78.4 (471) 20.6 (124) 0.3 (2) 0.0 (0) 0.0 (0) 0.7 (4)	73.8 (357) 25.2 (122) 0.4 (2) 0.0 (0) 0.2 (1) 0.4 (2)	73.2 (341) 25.8 (120) 0.4 (2) 0.2 (1) 0.0 (0) 0.4 (2)	75.4 (1,169) 23.6 (366) 0.4 (6) 0.1 (1) 0.1 (1) 0.5 (8)
Area you live in % (n)				
- Capital city/suburbs surrounding - Regional city - Regional area (e.g. small town) - Remote	42.8 (256)* 32.8 (196) 23.7 (142)* < 5 (< 5)*	15.0 (71)* 20.5 (97)* 59.2 (280)* 5.3 (25)	24.4 (113)* 33.7 (156) 37.4 (173)* 4.5 (21)	28.7 (440) 29.3 (449) 38.8 (595) 3.3 (50)
Highest level of education % (n)				
- Primary school - High school - Trade or apprenticeship - Undergraduate degree - Postgraduate degree	1.0 (6) 23.0 (138) 21.2 (127) 23.5 (141)* 31.2 (187)*	3.1 (15)* 20.1 (97) 21.9 (106) 29.8 (144) 25.1 (121)	< 5 (< 5) 22.8 (105) 19.3 (89) 33.0 (152)* 24.3 (112)*	1.6 (24) 22.0 (340) 20.9 (322) 28.3 (437) 27.2 (420)
Employment status % (n)				
- Retired - Working part-time - Working full-time - Unemployed	80.1 (479) 12.9 (77) 6.2 (37) 0.8 (5)	83.0 (401) 12.4 (60) 4.3 (21) < 5 (< 5)	81.9 (379) 12.5 (58) 4.8 (22) < 5 (< 5)	81.5 (1259) 12.6 (195) 5.2 (80) 0.6 (10)
Living status % (n)				
- Live alone - Live with spouse/partner - Live with other family - Live with friends - Live with others	35.5 (213) 53.0 (318) 9.8 (59) < 5 (< 5) 1.0 (6)	31.1 (150) 62.5 (302) 5.6 (27) < 5 (< 5) < 5 (< 5)	33.9 (157) 57.5 (266) 6.9 (32) 1.3 (6) < 5 (< 5)	33.6 (520) 57.3 (886) 7.6 (118) 0.7 (11) 0.7 (11)
Number of children M (SD) Range	2.4 (1.4)* 0–9	2.2 (1.3) 0–7	2.1 (1.2)* 0–7	2.2 (1.3) 0–9
Prescribed medication taking Range	3.0 (2.9) 0–25	2.9 (2.8) 0–22	3.2 (2.7) 0–20	3.0 (2.8) 0–25
Able to complete ADLs % (n)				
- Complete without difficulty - Some difficulty, need a little help - Moderate difficulty and need help - Significant difficulty need help	96.0 (575) 3.3 (20) < 5 (< 5) < 5 (< 5)	96.5 (467) 3.1 (15) < 5 (< 5) < 5 (< 5)	95.9 (446) 3.7 (17) < 5 (< 5) < 5 (< 5)	96.1 (1488) 3.4 (52) 0.5 (7) < 5 (< 5)
Help in the home (paid or family) % (n)				
- Yes - No	23.5 (141)* 76.5 (459)*	16.8 (81)* 83.2 (402)*	20.2 (94) 79.8 (372)	20.4 (316) 79.6 (1,233)

Note. *Denotes significant difference between regions. ADLs: Activities of Daily Living

Statistically significant differences were found between the UK-Ireland region and Canada-USA region for age, with the Canada-USA region being older. Significantly more participants from the Australia-NZ region lived in a capital city or the surrounding suburbs compared to the other two regions. Significantly more from Australia-NZ, Canada-USA lived in regional cities compared to the UK-Ireland and significantly more participants from the UK-Ireland lived in a regional area compared to the other two regions. The UK-Ireland and Canada-USA regions both had significantly more participants who had completed an undergraduate degree than the Australia-NZ region. Whereas post-graduate degree completion was reported significantly more often by Australia-NZ participants compared to the other two regions. The Australia-NZ region reported significantly more children than the Canada-USA region. Australia-NZ also had significantly more participants who had help in the home compared to the UK-Ireland region. No significant differences for sex, employment status, medications prescribed and ability to complete activities of daily living were found.

### Do older people set goals they would like to achieve?

Almost two-thirds (63.0%, *n* = 977) of the survey participants stated they set goals they wanted to achieve. The Australia-NZ (67.1%; *n* = 403) and Canada-USA (65.5%; *n* = 305) regions were significantly more likely to set goals than the UK-Ireland region (55.6%; *n* = 269), χ^2^(2, *n* = 1,551) = 16.875, *p* < 0.0001. Of the 63.0% who reported they set goals, 67.4% (*n* = 659) described their goals in an open-ended response. Many participants reported multiple goals within their response. In total, there were 1,773 goals themed, Australia-NZ averaged 1.66 per participant (*n* = 683 total number of goals), Canada-USA 1.42 per participant (*n* = 538), and UK-Ireland 1.12 per participant (*n* = 552) (see Table 1). UK-Ireland averaged significantly fewer goals per person than Australia-NZ and Canada-USA.

### Types of goals – overarching themes

Six overarching themes were identified from 946 goals reported, these are presented in Table 2. ‘Activities and experiences’ was the most frequently mentioned theme, followed by ‘health and wellbeing’ and ‘social connections and engagement.’ There was a statistically significant difference for ‘social connections and engagement’ with the UK-Ireland reporting these fewer times than participants from Australia-NZ and Canada-USA. Australia-NZ participants were significantly more likely to report ‘activities and experiences’ as a goal compared to Canada-USA and ‘home and lifestyle’ significantly more than both of the other regions. Canada-USA were significantly more likely to report goals around ‘finances and employment’ compared to the UK-Ireland.

**Table 2:** Overarching themes by region and sex

Over-arching Goals % (*N*)	Australia & NZ	UK & Ireland	Canada & the USA	Total
Health and Wellbeing	58.0 (220)	55.2 (139)	51.6 (146)	55.3 (505)
Social Connections and engagement	45.1 (171)	30.6 (77)	45.6 (129)	41.2 (377)
Activities and experiences	63.1 (239)	62.7 (158)	52.7 (149)	59.7 (546)
Finance and employment	15.3 (58)	9.9 (25)	18.4 (52)	14.8 (135)
Home and lifestyle	23.7 (90)	11.9 (30)	15.5 (44)	17.9 (164)
Attitude to life	14.0 (53)	13.1 (33)	13.1 (37)	13.5 (123)

χ^2^ (12, *N* = 914) = 51.611, *p* < 0.001			
Over-arching Goals % (*N*)	Female	Male	Total
Health and Wellbeing	58.4 (436)	41.7 (83)	54.9 (519)
Social Connections and engagement	41.0 (306)	44.2 (88)	41.6 (394)
Activities and experiences	59.8 (447)	55.8 (111)	59.0 (558)
Finance and employment	13.5 (101)	19.6 (39)	14.8 (140)
Home and lifestyle	19.8 (148)	11.1 (22)	18.0 (170)
Attitude to life	13.5 (101)	14.1 (28)	13.6 (129)

**χ**^**2**^**(6, ** ***N* = 946) = 32.184, *p* < 0.001**

Due to the small number of participants identifying as non-binary (*n* = 3), transgender (*n* = 1) and intersex (*n* = 1) the analysis only included those identifying as male or female for the differences between sexes questions. Females provided on average 2.06 responses and males 1.86. ‘Activities and experiences’, followed by ‘health and wellbeing’ and ‘social connections and engagements’ were the three highest reported goals respectively for females (see Table 2). Males reported ‘activities and experiences’, ‘social connections and engagements’ and then ‘health and wellbeing’ as their top three reported goals. There were significant differences between the sexes for ‘health and wellbeing’ and ‘home and lifestyle’ where it was more likely to be reported by females, whereas males were significantly more likely to report ‘finance and employment’ as part of their goals.

### Types of goals – sub-themes

**Fig. 1:**
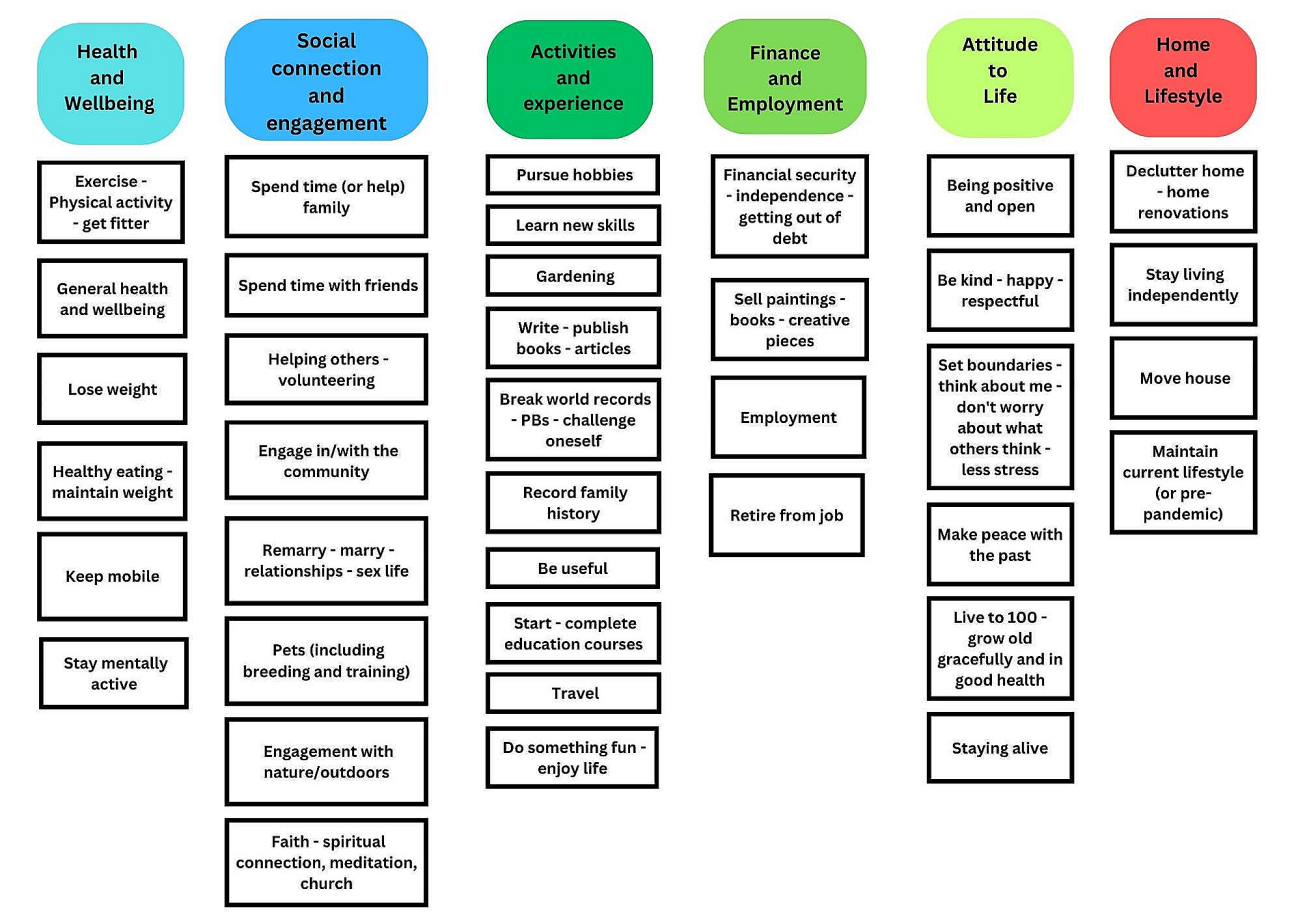
Conceptual map of the goals

The overarching themes were derived from 38 sub-themes, which are illustrated in Fig. 1 Conceptual map. ‘Activities and experiences’ included the largest number of sub-themes with 10 identified. Examples included hobbies, learning new skills, gardening, writing books, breaking records, being useful, travel and doing things that are fun. ‘Social connection and engagement’ included eight sub-themes, such as spending time with family and friends, volunteering, engaging with the community or outdoors and having a pet. ‘Health and wellbeing’ and ‘attitude to life’ both had six sub-themes, whereas ‘finance and employment’ and ‘home and lifestyle’ included four sub-themes respectively.

Table 3 presents the 38 sub-themes by region. The most prevalent sub-themes were exercise/physical activity/get fitter (35.6%), travel (28.6%), general health and wellbeing (22.6%) and spending time with family (or helping them) (20.6%). The Australia-NZ region was significantly more likely to report general health and wellbeing and gardening as important goals compared to both the UK-Ireland and Canada-USA. Australia-NZ and Canada-USA regions were also significantly more likely to state helping others and volunteering, and spending time with friends as goals compared to the UK-Ireland region. Healthy eating and maintaining weight was reported significantly more often by participants from Canada-USA than Australia-NZ. The Australia-NZ region was also significantly more likely to report spending time with family (or helping them) and stay living independently than participants living in the UK-Ireland region. Faith and spiritual connection, including going to church and meditating was reported significantly more often for Canada-US compared to both Australia-NZ and the UK-Ireland. However, the UK-Ireland were significantly more likely to state starting or completing educational courses and learning new skills as goals compared to those in the Canada-USA region. Finally, Canada-USA were more likely to report staying alive as a goal compared to the UK-Ireland.

**Table 3:** Sub-themes by region

Sub-theme Goals % (*N*)	Australia & NZ	UK & Ireland	Canada & USA	Total
**Health and Wellbeing**				
General Health & Wellbeing	28.6 (111)	16.8 (43)	19.6 (56)	22.6 (210)
Healthy eating and maintain weight	5.4 (21)	7.4 (19)	10.8 (31)	7.6 (71)
Exercise/physical activity/get fitter	36.3 (141)	39.8 (102)	30.8 (88)	35.6 (331)
Lose weight	9.0 (35)	9.0 (23)	9.1 (26)	9.0 (84)
Stay mentally active	5.7 (22)	4.7 (12)	4.9 (14)	5.2 (48)
Keep mobile	1.5 (6)	2.0 (5)	1.7 (5)	1.7 (16)
**Social connection and engagement**				
Helping others - volunteering	9.3 (36)	3.9 (10)	10.5 (30)	8.2 (76)
Spend time with family (or help them)	24.5 (95)	15.6 (40)	19.9 (57)	20.6 (192)
Faith and spiritual connection (meditation) church	2.3 (9)	2.0 (5)	7.3 (21)	3.8 (35)
Engage in/with the community	7.2 (28)	5.5 (14)	5.2 (15)	6.1 (57)
Spend time with friends	12.6 (49)	6.6 (17)	14.0 (40)	11.4 (106)
Pets (inc breeding, training)	1.8 (7)	2.0 (5)	2.4 (7)	2.0 (19)
Engagement with nature/outdoors	1.0 (4)	0.4 (1)	2.8 (8)	1.4 (13)
Remarry - marriage - new relationship - sex life	4.4 (17)	2.3 (6)	5.9 (17)	4.3 (40)
**Activities and experiences**				
Travel	30.9 (120)	25.8 (66)	28.0 (80)	28.6 (266)
Pursue hobbies	19.8 (77)	18.0 (46)	18.5 (53)	18.9 (176)
Starting or completing education courses	3.1 (12)	7.0 (18)	2.1 (6)	3.9 (36)
Learn new skills	11.6 (45)	15.6 (40)	7.0 (20)	11.3 (105)
Break world records - PBs - challenge oneself	2.1 (8)	5.5 (14)	3.5 (10)	3.4 (32)
Gardening	11.6 (45)	4.3 (11)	2.4 (7)	6.8 (63)
Do something fun - enjoy life	1.3 (5)	3.9 (10)	1.4 (4)	2.0 (19)
Be useful	2.8 (11)	2.3 (6)	3.1 (9)	2.8 (26)
Record family history	1.0 (4)	1.2 (3)	2.1 (6)	1.4 (13)
Write or publish book/s, articles	4.4 (17)	5.5 (14)	4.2 (12)	4.6 (43)
**Finance and employment**				
Financial security - independence - getting out of debt	7.7 (30)	4.7 (12)	9.8 (28)	7.5 (70)
Employment	5.4 (21)	3.5 (9)	5.9 (17)	5.1 (47)
Retire from job	1.3 (5)	0.8 (2)	1.0 (3)	1.1 (10)
Sell paintings, books, creative pieces	1.0 (4)	1.6 (4)	2.1 (6)	1.5 (14)
**Attitude to Life**				
Being positive and open	7.2 (28)	7.4 (19)	5.2 (15)	6.7 (62)
Be kind - happy - respectful	5.7 (22)	3.1 (8)	5.9 (17)	5.1 (47)
Live to 100 - grow old gracefully and in good health	3.6 (14)	3.5 (9)	1.4 (4)	2.9 (27)
Make peace with the past	1.5 (6)	1.2 (3)	0.3 (1)	1.1 (10)
Staying alive	2.6 (10)	0.8 (2)	5.6 (16)	3.0 (28)
Set boundaries - think about me - don’t worry about what others think - less stress	0.8 (3)	2.3 (6)	2.8 (8)	1.8 (17)
**Home and lifestyle**				
Stay living independently	7.5 (29)	2.0 (5)	3.5 (10)	4.7 (44)
Declutter home/home renovations	9.5 (37)	5.1 (13)	7.7 (22)	7.7 (72)
Move house	4.6 (18)	1.6 (4)	3.5 (10)	3.4 (32)
Maintain current lifestyle (or pre-pandemic)	1.8 (7)	2.0 (5)	1.7 (5)	1.8 (17)

**χ** ^**2**^ **(76,** ***N*** **= 930) = 194.426,** ***p*** **< 0.001**

Table 4 presents the sub-theme goals by sex, with 963 goals identified. Females identified exercise/physical activity/getting fitter (38.8%), travel (30.3%) and general health and wellbeing (22.3%) as their three most reported sub-theme goals. This was the same as the males, however only 23.8% of males reported exercise/physical activity/getting fitter as the most important sub-theme goal and 22.3% general health and wellbeing and travel, at equal second. Travel, healthy eating and maintaining weight, exercise/physical activity/getting fitter, losing weight, and learning new skills were all reported significantly more by females than males. Whereas males reported competing/breaking world records/challenging oneself, financial security/independence/getting out of debt, living to 100/growing old gracefully and in good health, remarrying/marry/relationships/sex life and staying alive significantly more often than females.

**Table 4:** Sub-themes by sex

Sub-theme Goals % (*N*)	Female	Male	Total
**Health and Wellbeing**			
General Health & Wellbeing	22.3 (169)	22.3 (46)	22.3 (215)
Healthy eating and maintain weight	8.6 (65)	3.4 (7)	7.5 (72)
Exercise/physical activity/get fitter	38.8 (294)	23.8 (49)	35.6 (343)
Lose weight	10.6 (80)	4.4 (9)	9.2 (89)
Stay mentally active	5.7 (43)	3.4 (7)	5.2 (50)
Keep mobile	1.6 (12)	1.5 (3)	1.6 (15)
**Social connection and engagement**			
Helping others - volunteering	20.9 (158)	19.9 (41)	20.7 (199)
Spend time with family (or help them)	8.7 (66)	7.3 (15)	8.4 (81)
Faith and spiritual connection (meditation) church	3.4 (26)	5.8 (12)	3.9 (38)
Engage in/with the community	6.6 (50)	4.9 (10)	6.2 (60)
Spend time with friends	12.2 (92)	7.8 (16)	11.2 (108)
Pets (inc breeding, training)	2.5 (19)	0.0 (0)	2.0 (19)
Engagement with nature/outdoors	1.3 (10)	1.5 (3)	1.3 (13)
Remarry - marriage - new relationship - sex life	2.9 (22)	9.7 (20)	4.4 (42)
**Activities and experiences**			
Travel	30.3 (229)	22.3 (46)	28.6 (275)
Pursue hobbies	18.6 (141)	18.9 (39)	18.7 (180)
Starting or completing education courses	4.0 (30)	2.4 (5)	3.6 (35)
Learn new skills	12.3 (93)	6.3 (13)	11.0 (106)
Break world records - PBs - challenge oneself	2.4 (18)	6.8 (14)	3.3 (32)
Gardening	7.0 (53)	5.3 (11)	6.6 (64)
Do something fun - enjoy life	2.1 (16)	1.9 (4)	2.1 (20)
Be useful	3.2 (24)	1.0 (2)	2.7 (26)
Record family history	1.7 (13)	0.5 (1)	1.5 (14)
Write or publish book/s, articles	4.4 (33)	3.9 (8)	4.3 (41)
**Finance and employment**			
Financial security - independence - getting out of debt	6.5 (49)	11.7 (24)	7.6 (73)
Employment	5.4 (41)	3.9 (8)	5.1 (49)
Retire from job	0.9 (7)	1.5 (3)	1.0 (10)
Sell paintings, books, creative pieces	1.2 (9)	2.4 (5)	1.5 (14)
**Attitude to Life**			
Being positive and open	6.5 (49)	7.3 (15)	6.6 (64)
Be kind - happy - respectful	5.3 (40)	4.9 (10)	5.2 (50)
Live to 100 - grow old gracefully and in good health	2.0 (15)	5.8 (12)	2.8 (27)
Make peace with the past	0.9 (7)	1.5 (3)	1.0 (10)
Staying alive	2.4 (18)	5.8 (12)	3.1 (30)
Set boundaries - think about me - don’t worry about what others think - less stress	1.8 (14)	1.9 (4)	1.9 (18)
**Home and Lifestyle**			
Stay living independently	5.9 (45)	0.0 (0)	4.7 (45)
Declutter home/home renovations	8.2 (62)	6.3 (13)	7.8 (75)
Move house	3.6 (27)	2.4 (5)	3.3 (32)
Maintain current lifestyle (or pre-pandemic)	2.1 (16)	1.0 (2)	1.9 (18)

**χ** ^**2**^ **(38,** ***N*** **= 963) = 129.462,** ***p*** **< 0.001**

## Discussion

This study found that the majority of older adults, living in different regions of the world, are setting personal goals. Six over-arching themes were identified from the data and not only included “health and well-being,” like much of the previous research in this area, but also an array of other factors that were identified as important in an older person’s life. Additionally, some of the themes aligned with the six determinants of active aging identified in the WHO’s Active Aging Policy [4], such as health, social, personal, and economic factors.

“Health and well-being” were identified by more than half of the participants in this study, which is similar to other studies exploring older adults’ personal goals. However, many of the sub-themes, such as physical activity, exercise, improving nutrition, controlling pain, or maintaining function, were also identified as higher, overarching themes within previous research [2, 17, 21]. This may be because fewer goals were described by participants in previous research or closed questions (tick boxes) were used in data collection. Also, unlike previous research, this current study included multi-country perspectives using the same open-answer questionnaire during the same time period, which may have increased the breadth of goals that were provided when compared to single country studies.

Working with older people to create future goals has been found to increase life satisfaction and quality of life [13]; thus, this may be a key way to intervene after a health issue arises. However, health interventions geared toward older people tend to give them “what is considered to be needed, but not what they want or hope for” [22, p. 299]. New ways to achieve goals that promote active aging continue to increase [2], and health care providers, social workers, and others may facilitate continued growth in this area by assisting older people in their goal setting and planning. For example, soliciting and focusing on self-defined goals when working with older adults can provide a sense of agency in creating a future that best supports and enhances their quality of life and overall well-being. Additionally, public health policies that aim to promote healthy aging could integrate elements of goal setting for older adults that focus on specific aspects of “activities and experiences” and “social connections and engagement” alongside health promotion and engagement. Those who work with older adults will need to shift their attitudes toward goal setting [2] by focusing their energy on self-determination and elevating the voices of older adults as well as moving beyond care goals. Arguably, health goals are essential to much of this work, but a holistic approach is needed to improve subjective well-being and overall life satisfaction and to build trust and rapport.

“Activities and experiences” was the most prevalent theme across the six regions, which have been described in previous research under the guise of leisure [22], cultural activities, volunteering, skill development [23], travel [21], and intellectual pursuits [2]. Yet none of this previous research covered the range of activities older people enjoy and how it differs depending on the country or sex of the participants. Nor have they illustrated that “activities and experiences” are perceived as more important as personal goals than “health and well-being” or “social connections and engagement” by older adults. This research illustrates the importance of “activities and experiences” for older adults and that they want to continue participating, and in some cases, achieving in activities they enjoy (e.g., breaking world records). Health professionals could consider discussing whether these are of importance to their older patients, and if so, linking their health and rehabilitative goals to the “activities and experiences” of their choice. This approach is often described in the reablement literature. Reablement being defined broadly as a person-centered approach aimed at enhancing a person’s physical and/or other functioning to increase or maintain independence in meaningful activities of daily living [24]. However, goals are often focused around health, function, or social connections rather than “activities or experiences” [24, 25].

“Social connections and engagement” were also identified in the top three themes for both sex and region. Females in this current study placed greater emphasis on “health and well-being” (second highest) as a goal, whereas males identified “social connections and engagement” as more important than their “health and wellbeing” goals. This may be due to women already prioritizing “social connections and engagement” [26] and therefore not feeling a need to include it as a goal. Similarly, fewer males noted exercise or physical fitness as a priority, yet research shows older age males are often more physically active than females [27]. Thus, they may not include this as a personal goal if it is already being achieved. However, this is outside the scope of this study, and it is not possible to be certain on the reasons why these goals were prioritized by the different sexes. Focus groups or interviews could facilitate greater understanding of how gender influences goal-setting.

“Finance and employment” were perceived as important to participants in the Australia/New Zealand and Canada/USA regions, but not as much in the UK/Ireland. It is difficult to determine why this may be the case, but potentially the different pension systems and health and social care systems may play a role. Males were also significantly more likely to include financial security, independence, and getting out of debt as goals compared to females. This study did not explore reasons why, and we are therefore unable to speculate as to why this was the case. Perhaps traditional gender role socialization plays a role in personal goal setting. “Finances and employment” were not commonly described in previous research looking at personal goals of older adults, but it has been described within zero-sum, extrinsic frameworks [28, 29]. Future research is needed to examine these connections, generationally and by gender identity.

The Australia/NZ region were significantly more likely to include “home and lifestyle” goals than both the UK/Ireland and Canada/USA regions. Also, the Australia/NZ region had almost twice the proportion of participants living in a capital city or regional city compared to the other two regions and far fewer in regional and remote areas. Study participants were not asked whether they owned their own home or rented, and this may have provided one reason as to why it was more of a goal for some than others. Other studies exploring personal goals of older adults rarely included “home and lifestyle” factors within their work. This is an area ripe for further examination as it relates to aging in place and what meaning the home has for older people as they age.

Enjoying life and having a positive attitude to life has been included in previous goals research for older adults [2, 23], and like this current study, it was not of the highest priority when setting their goals. However, having a positive attitude, enjoying life, and being kind and respectful is viewed as important enough by older people to be included in their personal goals. Educating health care providers, social workers, and others who primarily work with older people through continuing education programs run by their disciplines (e.g., Australian Physiotherapy Association), online courses or webinars (national societies e.g., Gerontological Society of America) would allow them to think about goal setting beyond specific health care needs and promote greater generativity and well-being.

There are a number of strengths of this study, including open questions with no examples provided for types of goals to include, therefore reducing the opportunity for bias and only capturing the thoughts of the participants. Collecting data across multiple countries simultaneously using the same questionnaire has, to our knowledge, not been conducted previously in personal goal setting and provides an opportunity to directly compare perspectives of older people across three regions of the world. Multi-country studies have been undertaken previously, but they linked data between countries after data collection was completed, and often the questions being asked were not worded exactly the same across the different countries. Limitations included only involving English speaking countries in the questionnaire and predominantly including people with access to the internet and who were Facebook users. There was also an over-representation of females and ethnicity and income were not collected. There is also a chance that how we described where someone was living may have been misinterpreted between capital city and surrounding suburbs and how this is described across the different countries included in the study. We also combined countries to explore the three regions and there may have been differences between the countries, for example with USA and Canada having different healthcare systems.

## Conclusions

This study supports the notion that older adults can and do set personal goals and that they are wide ranging. Goal setting is prevalent in health care, but research often states from the health professionals’ perspective that it is difficult to identify goals or that older adults either do not like or find it difficult to set goals [30]. Perhaps other factors, such as the approach to identifying these goals, the way the question about goal setting is phrased, or lack of time given to the older person to think about their goals are greater issues than their ability to identify them. Health professionals may like to consider different methods for elucidating this important information from their patients that solicits self-defined goals and includes programming that factor in elements of activities and experiences and social connections and engagement.

## Data Availability

The datasets used and/or analysed during the current study are available from the corresponding author on reasonable request.

## Abbreviations

NZ: New Zealand
SPSS: Statistical Packages for the Social Sciences
UK: United Kingdom
USA: United States of America
WHO: World Health Organization
